# In Situ Scanning Transmission Electron Microscopy/Transmission Electron Microscopy Study of Defect-Driven Ag Ion Dynamics and Filament Evolution in CuO Nanowire-Based Memristors

**DOI:** 10.1021/acsami.5c21065

**Published:** 2026-01-05

**Authors:** Ching-Heng Hung, Chong-Chi Chi, Kai-Yuan Hsiao, Ming-Yen Lu

**Affiliations:** a Department of Materials Science and Engineering, 34881 National Tsing Hua University, Hsinchu 300, Taiwan; b Instrumentation Center, 34881 National Tsing Hua University, Hsinchu 3000, Taiwan

**Keywords:** resistive switching, CuO nanowires, in situ TEM, electrochemical metallization processes, ion migration, conduction mechanisms

## Abstract

Memristor-based technologies are pivotal for advancing in-memory computing and neuromorphic systems, addressing the von Neumann bottleneck by enabling low-power, high-density data storage. This study investigates the resistive switching (RS) behavior of p-type CuO nanowires (NWs) synthesized via thermal oxidation of a Cu foam integrated with Ag (active) and Au (inert) electrodes. In situ transmission electron microscopy (TEM) and scanning TEM (STEM) reveal the dynamic formation and dissolution of Ag-based conductive filaments under an electrical bias, driven by electrochemical metallization (ECM). The CuO NWs exhibit unique axial planar defects that facilitate Ag^+^ ion migration and nucleation, enhancing RS performance. Electrical measurements demonstrate volatile and nonvolatile switching transitions modulated by compliance current, with asymmetric Ag/CuO NW/Au devices showing diode-like behavior due to Schottky barrier modulation. Conduction mechanisms, including Schottky emission, space-charge-limited current, and Poole–Frenkel emission, are elucidated, transitioning to ohmic conduction in the low-resistance state. These findings provide critical insights into defect-mediated filament dynamics and electrode-dependent RS mechanisms, advancing the development of CuO NW-based memristors for next-generation neuromorphic computing applications.

## Graphical abstract

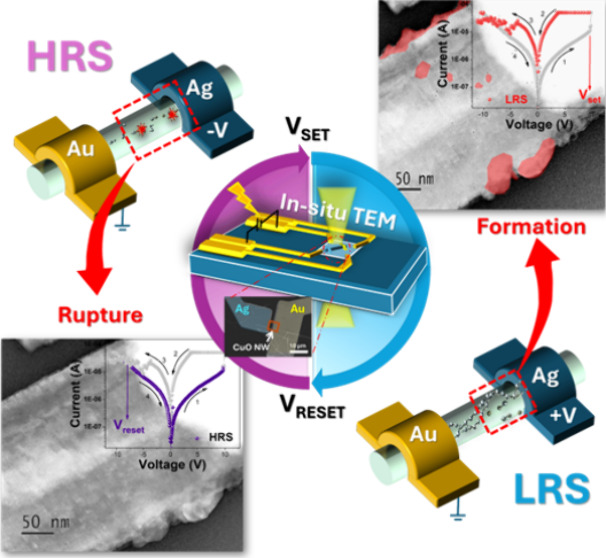

## 1 Introduction

Recent advancements in artificial intelligence (AI) technology have substantially increased the demand for high-speed computing while emphasizing the need for reduced power consumption in electronic devices.^1^ The von Neumann bottleneck, caused by frequent data transfer between discrete memory and processing units, limits computational efficiency and increases energy consumption.^2^ Memristor-based technologies address this challenge by enabling in-memory computing, thereby enhancing both computational speed and energy efficiency.^3^ Consequently, increasing research efforts are being directed toward the development of neuromorphic computing, which aims to emulate the functional mechanisms of biological neurons and synapses in the human brain.^4,5^ With low-power consumption and scalability, memristors with metal/insulator/metal (MIM) structures are gaining attention due to their simple structure and rapid read/write capabilities, these advantages make memristors promising candidates for next-generation memory.^6^

Memristors, with both volatile and nonvolatile switching characteristics, present significant potential for high-density data storage and neuromorphic computing systems.^7^ Nonvolatile memories (NVMs) typically require higher voltages and longer writing times compared to volatile memories (VMs). VM devices, which automatically return to their initial state, have also garnered significant attention in recent studies. The electrical performance of these devices is influenced by carrier diffusion within the electric field, making them susceptible to structural defects such as lattice imperfections, material interfaces, impurities, and grain boundaries. From this perspective, in situ TEM provides valuable insights into the dynamic changes of the materials, offering reliable evidence for studying filament growth behavior and underlying mechanisms.^8,9^

In recent years, investigations into RS have mainly focused on vertically stacked MIM structures.^10^ 1D NWs offer improved scalability and integration density, along with precise control and easier observation of electron transport within individual NWs, making them superior candidates as memristor materials.^11^ The high aspect ratio (length significantly greater than width) of 1D NWs enhances surface interaction and provides superior scalability and integration density,^12,13^ enabling precise control and easier observation of electron transport within individual NWs. Among 1D NWs, p-type CuO NWs have garnered significant attention due to their tunable electrical properties and inherent structural defects,^14^ which can enhance CF formation and RS dynamics. Unlike conventional thin-film memristors, CuO NWs offer a unique platform for studying filament growth at the nanoscale, particularly when coupled with advanced characterization techniques such as in situ transmission electron microscopy (TEM) and scanning TEM (STEM).^15^ These techniques provide real-time, atomic-scale insights into the dynamic processes of ion migration and filament evolution under electrical bias, offering a deeper understanding of the underlying electrochemical metallization (ECM) and valence change mechanisms (VCM).^8^ In this context, the integration of CuO NWs with active (e.g., Ag) and inert (e.g., Au) electrodes enables the exploration of electrode-dependent RS behaviors, shedding light on the interplay between the material properties and device performance.

We report p-type CuO NWs synthesized via thermal oxidation of a Cu foam substrate, featuring unique axial planar defects that facilitate conductive filament formation in the present study.^16^ Using in situ TEM/STEM, we provide direct, time-resolved evidence that links the microstructural dynamics of Ag species redistribution and filament evolution in a single-NW device directly to its RS behavior. Specifically, the intrinsic hole-transport nature of p-type CuO and its characteristic TB provide a unique platform to investigate how hole conduction and Ag diffusion jointly govern switching kinetics. By tracking the ionization, migration, and clustering of Ag species in real time, we elucidate the defect-guided filament dynamics driving HRS–LRS transitions, offering valuable insights for designing reliable 1D memristive and neuromorphic systems.

## 2 Results and Discussion

### 2.1 Morphological and Structural Characterizations of CuO NWs

Large-scale, vertically aligned CuO NWs were synthesized via direct thermal oxidation^14,17^ of commercial Cu foam substrates, as detailed in the Experimental Methods section. The cross-sectional SEM image in Figure 1a reveals densely packed CuO NWs oriented perpendicular to the substrate surface, exhibiting uniform diameters and high aspect ratios across the oxidized region. Individual NWs observed at higher magnification (Figure 1b) show a diameter of approximately 100 nm, and additional SEM images in Figure S2 confirm conformal coverage across the porous Cu foam, indicating the scalability and structural uniformity of the synthesis method. Thermal oxidation of Cu substrates results in a self-organized bilayer structure comprising thermodynamically stable Cu_2_O and CuO phases. Thermodynamic driving forces establish a bioxide stratification during Cu oxidation: high oxygen activity at the gas/oxide surface stabilizes CuO, whereas lower activity at the buried interface favors Cu_2_O, producing a CuO/Cu_2_O bilayer controlled by outward Cu-cation diffusion. The interfacial CuO to Cu_2_O conversion generates a molar-volume mismatch that builds compressive stress in the outer CuO, and the stress creates an additional chemical-potential gradient that accelerates grain-boundary cation transport toward the surface, where incorporation at step and kink sites nucleates and sustains CuO NW growth.^18^ In the initial stage of oxidation, Cu is first oxidized into an intermediate Cu_2_O layer. With prolonged exposure to elevated temperatures and an oxygen-rich environment, the Cu_2_O layer is further oxidized to form a CuO overlayer. In parallel, a dense array of CuO NWs spontaneously emerges on the surface, driven by anisotropic oxidation kinetics.^18,19^ This vertically stratified architecture enhances structural integrity and provides a robust platform for NWs growth.^20^ TEM analysis in Figure 1c reveals a longitudinal grain boundary extending parallel to the axial direction of CuO NWs, which is a typical feature of CuO NWs in the present study (Figure S3). The longitudinal defects in NW are inherently associated with their growth mechanism, Cu ions diffuse from the base to the tips and then oxidize during the thermal oxidation process. This diffusion-driven growth results in the presence of grain boundaries and defects within the CuO NWs.^20^ Although a rigorous statistical distribution of the NW dimensions is not presented, SEM and TEM surveys over large areas indicate a uniform growth trend across the substrate. The high-magnification SEM image in Figure S3, the NWs maintain a uniform diameter (∼100 nm). Furthermore, the TEM analysis confirms that individual NWs share consistent microstructural features, such as the axial twin boundaries (TBs). The devices also exhibit highly reproducible electrical characteristics with negligible device-to-device variation. This robust functional consistency strongly suggests that the morphologically observed uniformity is sufficient to ensure reproducible device performance.

**Figure 1:**
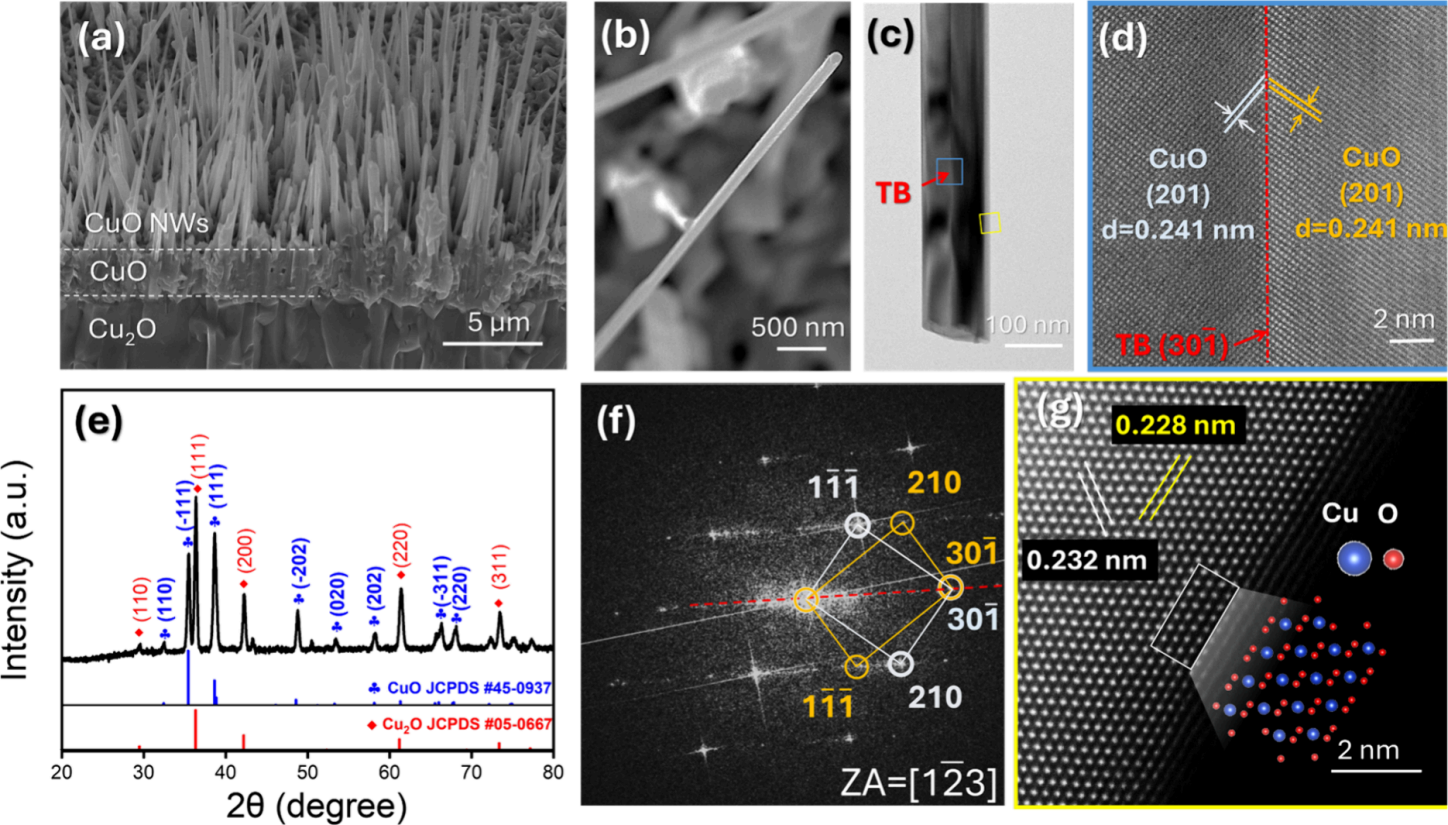
Fabrication of CuO NWs on the Cu foam substrate. (a) SEM image of CuO NWs on the Cu foam substrate, showing uniform, wire-like morphology. (b) High-magnification SEM image of single CuO NW, exhibiting a diameter of ∼100 nm. (c) TEM image of a CuO NW, where a longitudinal contrast feature is visible along the NW axis, indicative of an internal planar defect (twin boundary (TB)). (d) HRTEM image of the blue square region in panel (c), showing the grain boundary separates two different sets of the lattice fringes. (e) XRD pattern of the sample. (f) The corresponding diffraction pattern of twining structure in panel (d), acquired along the [1 2̅ 3] zone axis. (g) HAADF HRSTEM image of the CuO NW, the schematic shows the atomic arrangements of CuO along [011], where blue and red spots represent Cu and O atoms.

A distinct contrast region of a planar defect is present in Figure 1c, further magnified in Figure 1d, where high-resolution TEM (HRTEM) confirms a grain boundary separating two crystallographic grains. X-ray diffraction (XRD) analysis using Cu Kα radiation (λ = 0.15406 nm) is presented in Figure 1e, and sharp and well-defined characteristic diffraction peaks are indexed to Cu_2_O (JCPDS No. 05–0667) and monoclinic CuO (JCPDS No. 45–0937). The oxidation process also results in the formation of a distinct bilayer structure: a Cu_2_O layer adjacent to the metallic Cu substrate and a top CuO NW layer, as confirmed by XRD and cross-sectional SEM imaging.^18^ The results demonstrate the effectiveness of thermal oxidation in the production of high-quality CuO NWs. The selected-area electron diffraction (SAED) pattern in Figure 1f corresponds to the twinned structure in Figure 1d, showing two identical sets of diffraction spots with the rotation angle of 72.49°, which is the typical feature of the twin structure. The diffraction spots are indexed to be (1 1̅1̅), (30 1̅), and (210) of CuO along the [1 2̅ 3] zone axis, with symmetric diffraction features observed from both crystallographic domains, and reveal that the mirrored diffraction spots relationship confirms the twin structure. The HAADF HRSTEM image in Figure 1g reveals the atomic arrangements of CuO NWs. The contrast in HAADF-STEM imaging is tied to the atomic numbers (Z) of the elements; atoms with higher Z numbers produce a greater signal contrast in the STEM images.^21,22^ Due to the difference in Z numbers between Cu and O, Cu atoms appear as bright spots in the image, while O atoms are hardly visible. The inset depicts the atomic model of monoclinic CuO with Cu and O atoms represented by blue and red dots, respectively. Moreover, the interplanar spacings of 0.223 and 0.228 nm correspond to the (1 1̅ 1) and (200) planes of monoclinic CuO, respectively. The high crystallinity and well-defined twin interfaces provide a favorable platform for investigating filament formation and defect-modulated RS behavior in subsequent in situ electrical experiments.

### 2.2 Device Design and Characterization

To facilitate in situ TEM observation of structural dynamics under electric field stimulation, the NWs were deposited onto a homemade TEM chip incorporating a Si_3_N_4_ membrane window. A homemade Si_3_N_4_ chip (E-chip) was designed to interface with the Protochips Fusion holder, with the Si_3_N_4_ window serving as the TEM/STEM viewing window. A step-by-step schematic of the TEM Si_3_N_4_ chip fabrication flow is presented in Figure S5. Schematic illustrations are presented in Figure 2a with the SEM image of the device in Figure 2b. Electrode preparation was carried out using photolithography with a maskless digital light processing (DLP) system and electron beam evaporation of metal electrodes. This process enabled the deposition of two metal electrodes at the ends of the NWs, forming a 1D metal/insulator/metal (1D MIM) configuration. In order to better understand the respective roles of active Ag and inert Au electrodes in governing the mechanism and dynamics of metallic filament formation, three distinct electrode configurations, Ag/CuO NW/Ag, Au/CuO NW/Au, and Au/CuO NW/Ag with an interelectrode spacing of 2 μm, were utilized. The TEM image of the device reveals the Ag electrode on the left and the Au electrode on the right, connected by CuO NWs. Corresponding EDS elemental mapping, shown in Figure 2c, confirms the spatial distribution of Ag, Au, Cu, and O elements, providing a reliable reference for subsequent analysis of material migration during cyclic device operation. The use of asymmetric electrodes enables direct visualization of the active electrode behavior under bias. Focusing on the CuO NW adjacent to the active Ag contact enables unambiguous resolution of the Ag/CuO switching mechanism. In this zone, the appearance and subsequent growth/coalescence of Ag NPs are directly observed. In the following sections, we investigate the RS behavior and the evolution of CuO NW-based devices under in situ electrical stimulation, with particular emphasis on the correlation between Ag migration and the underlying switching mechanisms.

**Figure 2:**
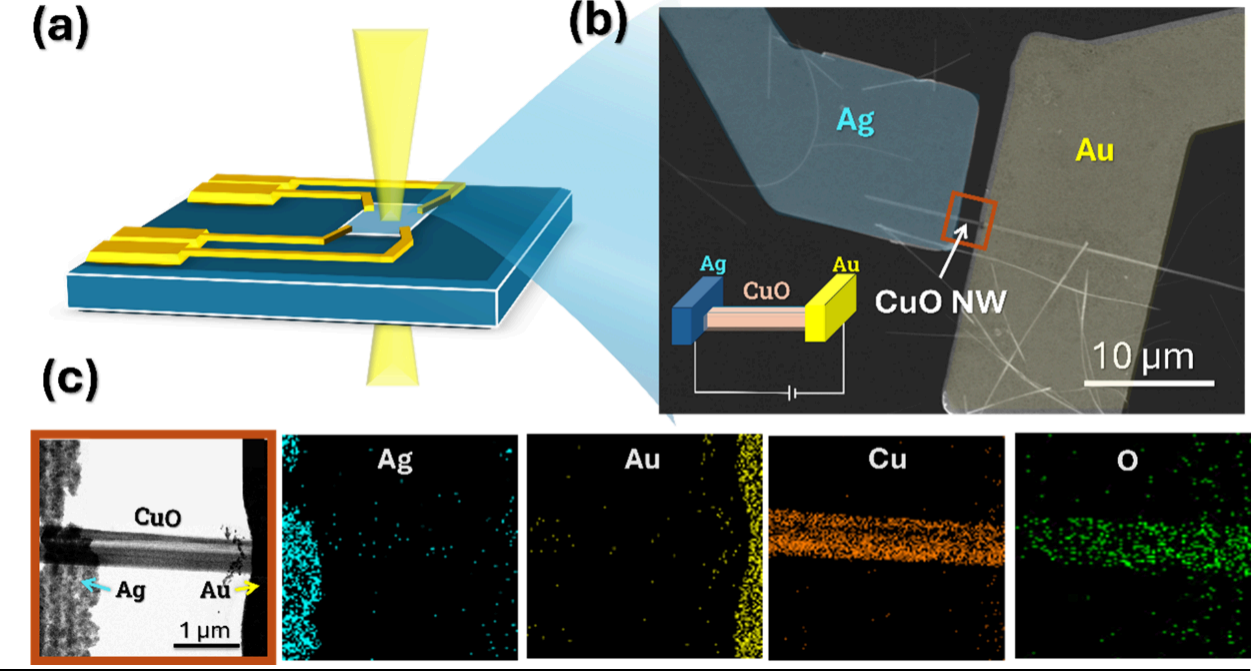
(a) Schematic illustration of the preparation of the Ag/CuO NWs/Au structure on a homemade Si_3_N_4_ chip designed for in situ TEM observation. (b) SEM image of the fabricated device showing a single CuO NW bridging Ag and Au electrodes. The inset displays the schematic of the device. (c) TEM image of the Ag/CuO/Au device along with corresponding EDS elemental mappings of Ag, Au, Cu, and O signals.

### 2.3 Electronic Conduction and Mechanism

The electrical characteristics of CuO NW-based memristive devices with different electrode configurations, Ag/CuO NW/Ag (symmetric active electrodes), Au/CuO NW/Au (symmetric inert electrodes), and Au/CuO NW/Ag (asymmetric electrodes), are shown in Figure 3a–c, respectively. All measurements were conducted under DC voltage sweeping with the *I*_CC_ set to 1 μA. Figure 3a displays the *I*–*V* results of the symmetric Ag/CuO NW/Ag configurations; a sharp increase in current is observed at threshold voltages (*V*_TH_) of approximately +4.8 and −5.0 V followed by an abrupt return to the high resistance state (HRS) once the voltage bias is removed. Polarity-independent switching and the lack of retention confirm volatile threshold-type behavior. The observed conductance modulation is associated with an ECM mechanism^23^ wherein Ag ions migrate into the CuO NW under an electric field, forming transient CFs that collapse immediately upon field removal. This reversible formation and dissolution of CFs suggest that both electrodes actively participate in filament formation. In contrast, the Au/CuO NW/Au device, composed of two inert electrodes, exhibits negligible RS behavior even under a large voltage sweep ranging from −30 to +30 V, as shown in Figure 3b. The absence of switching events indicates that in the absence of electrochemically active species such as Ag ions, filament formation or valence modulation is suppressed. These findings imply that the valence change mechanism (VCM), typically driven by oxygen vacancy dynamics, is not effectively triggered in the CuO NW geometry for inert contacts. This result highlights the critical role of active metal electrodes in enabling the ECM-type switching behavior in 1D systems. The limited switching behavior can be attributed to two factors: (i) inadequate oxygen vacancy generation and redistribution at the Au/CuO interface^24^ and (ii) the absence of redox-active species necessary for filament formation,^25^ collectively hindering effective ECM dynamics. Figure 3c shows the *I*–*V* characteristics of the asymmetric Ag/CuO NW/Au device over 30 consecutive bipolar voltage sweep cycles (0 V → + 8 V → – 8 V → 0 V). Under a positive bias applied to the Ag electrode, the device exhibits pronounced volatile RS, whereas negligible current modulation is observed under reverse bias. The current increases abruptly at a threshold voltage (*V*_TH_) of approximately +4.8 V, followed by a rapid decay at a hold voltage (V_H_) near +0.5 V, implying a threshold-type switching characteristic. The observed rectification characteristics exhibit 2–3 orders of magnitude current enhancement under positive bias. The *I*–*V* curve displays that the current under positive bias is significantly higher than that under negative bias. The work function mismatch between Ag (∼4.3 eV) and p-type CuO (∼5.3 eV) leads to the formation of a Schottky barrier at the Ag/CuO interface, which undergoes dynamic modulation under bias, further enhancing the forward current injection and enabling field-driven Ag migration, demonstrating diode-like behavior under forward-bias conditions. We further investigate the evolutions of switching behavior under various *I*_CC_ levels ranging from 10 nA to 100 μA under positive bias, as shown in Figure 3d. Notably, the RS behavior progressively shifted from VM to NVM as *I*_CC_ exceeded 10 μA, a transition intricately linked to the stability of the CF. At low *I*_CC_ (e.g., ≤ 10 μA), the device exhibits volatile switching characteristics. As the *I*_CC_ increases higher than 10 μA, the device exhibits a clear transition from volatile to nonvolatile switching behavior. At higher *I*_CC_ levels, the *I*–*V* curves display stable hysteresis loops without abrupt current drops at 0 V, indicating the formation of robust CFs with enhanced retention. This *I*_CC_-dependent modulation suggests that greater current compliance promotes more extensive Ag ion reduction and filament consolidation, thereby improving filament persistence and switching stability.

**Figure 3:**
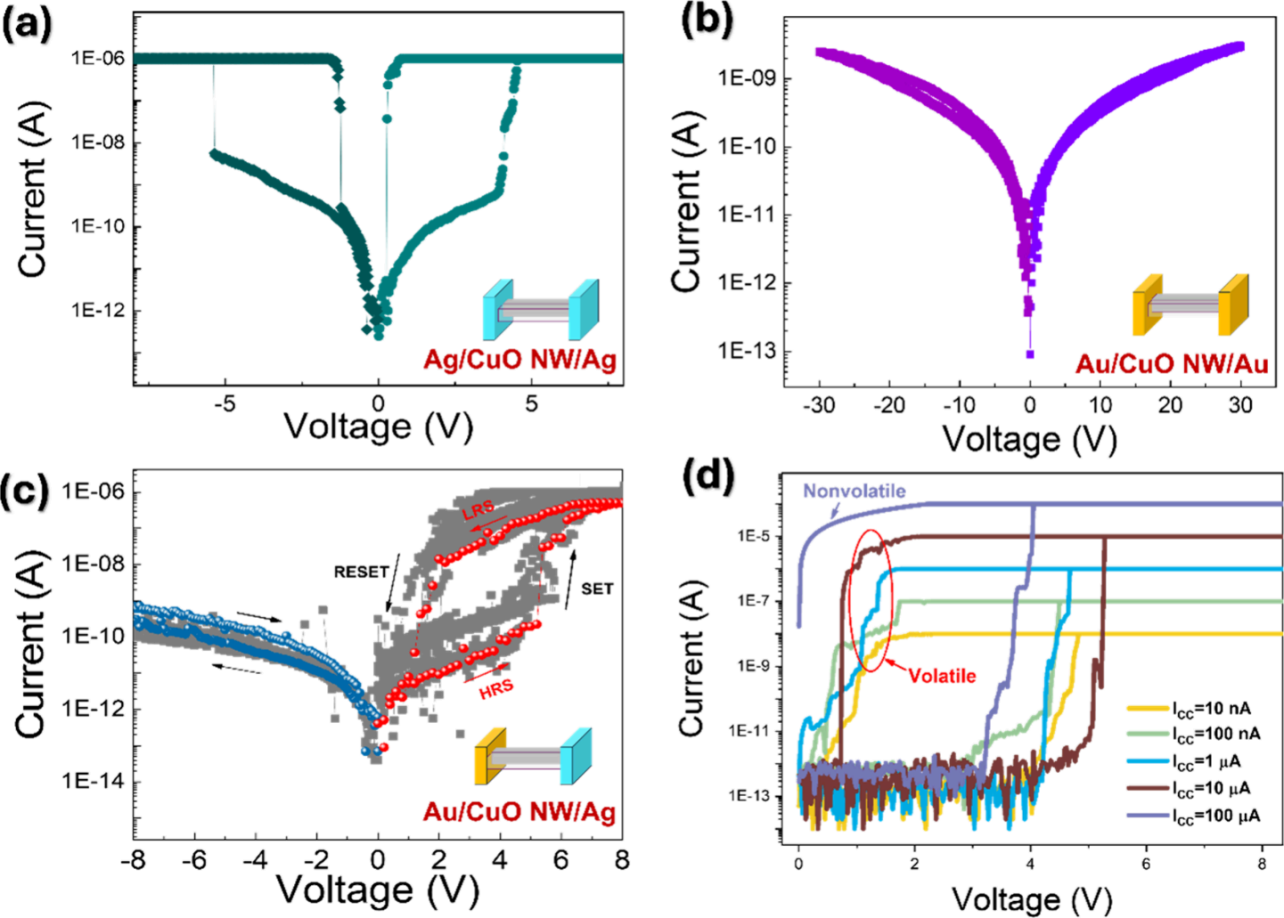
RS switching characteristics of CuO NW-based devices with different electrode configurations. (a) *I*–*V* curves of the symmetric Ag/CuO NW/Ag device showing bipolar volatile threshold switching, (b) *I*–*V* curves of the symmetric Au/CuO NW/Au device exhibiting negligible switching behavior, and (c) *I*–*V* curves of the asymmetric Au/CuO NW/Ag device revealing diode-like volatile RS behavior. (d) *I*–*V* curves of the Au/CuO NW/Ag device under positive bias with different compliance current (*I*_CC_) settings, demonstrating tunable multilevel switching behavior. The transition from the volatile to nonvolatile regimes occurs as *I*_CC_ increases.

In order to gain a more comprehensive understanding of the CF formation and switching dynamics in Ag/CuO NW/Au memristive devices, a systematic examination of the current density–electric field (*J*–*E*) characteristics was performed. The results in Figure 4a highlight the fundamental structural dynamics governing RS behavior. The J–E plot, presented as ln­(*J*) versus ln­(*E*), clearly distinguishes the dominant conduction mechanisms operating at different electric field regimes. Notably, the linear slope (∼1) observed in the low-resistance state (LRS) is indicative of ohmic conduction, consistent with the presence of continuous CF bridging the electrodes. The HRS, however, exhibits varying slopes. Schottky and Poole-Frenkel fitting applied to the *J*–*E* curve reveal the dominant conduction mechanisms under different electric fields. After the abrupt SET transition, the current exhibits a plateau that is determined by the externally imposed compliance current *I*_cc_, which is applied to prevent hard breakdown of the Ag/CuO NW/Au device. Thus, the apparent saturation in the LRS reflects instrumental current limiting rather than intrinsic saturation of the CF, and the ohmic behavior discussed in Figure 4a is evaluated from the low-field region below the compliance limit. The schematic diagram in Figure 4b shows the energy band structure of Ag/CuO NW/Au before contact, revealing the energy levels of Ag, CuO, and Au. At the initial stage, conduction is primarily governed by Schottky emission (Figure 4c). In this regime, hole injection is restricted by thermionic emission across the metal–semiconductor interface, which is influenced by the interfacial barrier height arising from the mismatch in work functions between Ag (∼4.26 eV) and p-type CuO (∼5.2 eV). As the electric field increases, the conduction mechanism shifts to SCLC, as depicted in Figure 4d. In the intermediate regime, the injected holes tend to accumulate in the trap states that are intrinsically present in CuO NWs formed via thermal oxidation. This accumulation can result in a high density of defects, which may further trap holes under high voltage conditions, ultimately leading to space-charge accumulation and the nonlinear conduction behavior characteristic of SCLC.

**Figure 4:**
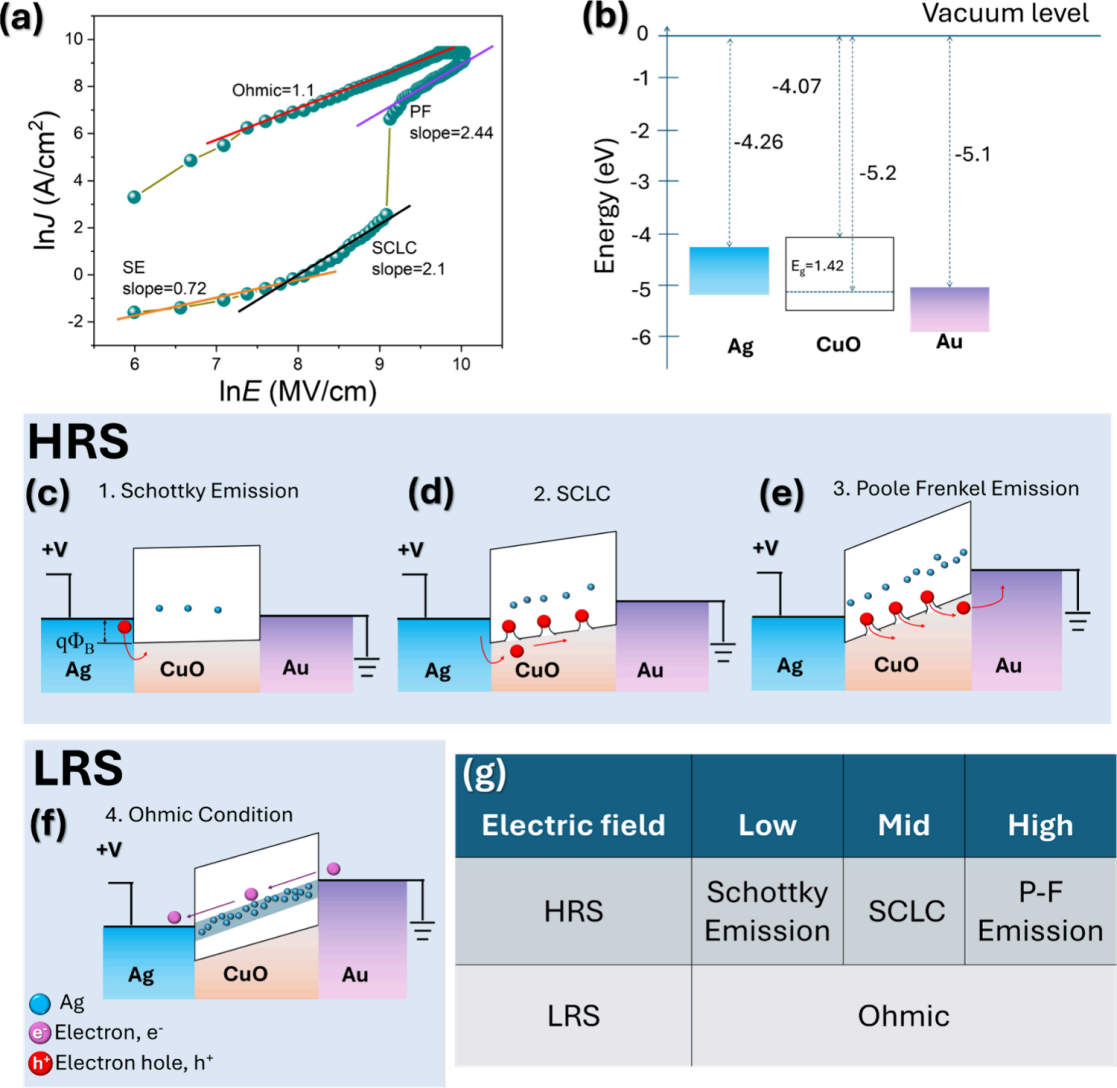
Investigation of the conduction mechanisms in the asymmetric Ag/CuO NW/Au device. (a) Plot of ln­(*J*) versus ln­(*E*) for the positive bias region of RS. (b) Schematic diagram of the energy band of the Ag/CuO NW/Au device prior to contact. (c–e) Energy band for HRS governed by Schottky emission, SCLC, and Poole–Frenkel emission mechanisms, respectively. (f) Energy band for LRS characterized by ohmic conduction. (g) Summary of different conduction mechanisms.

At elevated electric fields, conduction is primarily governed by the Poole–Frenkel emission mechanism (Figure 4e). In this regime, the strong electric field lowers the potential barriers around hole traps, facilitating their release into the valence band and thereby enhancing the charge transport. This field-assisted detrapping is particularly significant in defect-rich CuO NWs, where numerous trap states exist due to thermal oxidation. Concurrently, when the electric field reaches a sufficiently high threshold, Ag^+^ ions begin to migrate from the Ag electrode into the CuO NW matrix. These mobile ions are subsequently reduced to metallic Ag atoms, initiating the ECM process. The reduced Ag atoms nucleate into discrete nanoparticles (NPs), which grow and coalesce along energetically favorable pathways—typically along grain boundaries (GBs) or other extended defects. As these metallic Ag clusters continue to grow, they eventually form a continuous or nearly continuous filament, resulting in the device switching into an LRS. In this state, charge transport becomes dominated by ohmic conduction due to the presence of the highly conductive metallic filament. This filament effectively bypasses the trap-limited conduction pathways of the semiconducting CuO NW, as reflected by the linear *I*–*V* characteristics observed in the LRS and illustrated in the band diagram (Figure 4f). Consequently, the conduction mechanism undergoes a distinct transition—from semiconductor-limited transport mechanisms (such as Schottky emission, SCLC, and Poole–Frenkel emission) to an ohmic regime characterized by linear current–voltage behavior and significantly enhanced conductivity. Figure 4g summarizes these mechanisms across different operational stages, providing a comprehensive view of the charge transport dynamics under varying electrical conditions. Together, these electrical and morphological findings offer compelling evidence supporting an ECM-type RS mechanism in memristive devices based on p-type CuO NWs.

### 2.4 Dynamic Mass Transfer of Resistive Switching in CuO NWs

We conducted in situ TEM during electroforming and RS processes; this configuration enhances the potential for capturing filament dynamics within the TEM field of view. The stability of the CF is dependent on the external bias and proportional to the *I*_CC_.^26^ Typically, the *I*_CC_ is carefully optimized to maintain the memristor device within a “soft breakdown″ regime, characterized by a controlled and localized disruption of the oxide’s insulating matrix instead of an irreversible dielectric failure. This operational condition facilitates the formation of nanoscale CFs via electric field-induced ion migration or defect generation while simultaneously minimizing damage to the dielectric layer. Operating within this regime ensures stable electrical characteristics and repeatable RS behavior—both of which are essential for the reliable function of memristor-based devices. The in situ STEM observation for the present study was carried out under mild conditions with the current density of 145 pA. The devices were fabricated through lithographically patterned electrodes on an E-chip, and no structural change and damage of the materials were observed under electron-beam illumination without bias, which collectively confirms that the electron beam may take less effect on the evolution of Ag. The device depicted in Figure 5a features a tunneling gap of 500 nm and NW diameter of 150 nm. Under zero bias, no Ag NPs are observed, and the CuO NW exhibits a smooth, uniform morphology, confirming its pristine structural integrity prior to electrical stress. The absence of Ag NPs under unbiased conditions further confirms that filament formation is strictly electric field-dependent—a key feature of the ECM mechanism. An enlarged TEM image of the region near the active Ag electrode, highlighted by the dashed green box in Figure 5a, provides additional structural insight. Using in situ TEM, the complete forming process was recorded and analyzed frame-by-frame to trace the appearance and evolution of Ag NPs on the CuO NW. Two specific regions, marked by yellow and red dashed boxes in Figure 5b, exhibit significant morphological changes under applied bias. Figure 5c presents the *I*–*V* characteristics of a Ag/CuO NW/Au device during the initial forming process under a *I*_CC_ of 50 μA, alongside corresponding in situ TEM images; states 1–4 represent the specific conditions during the forming process. Figure 5d,e presents a time-sequenced series of TEM images from the yellow-box and red-box regions in Figure 5b, revealing electric field-induced dynamic changes in Ag NPs distribution.

**Figure 5:**
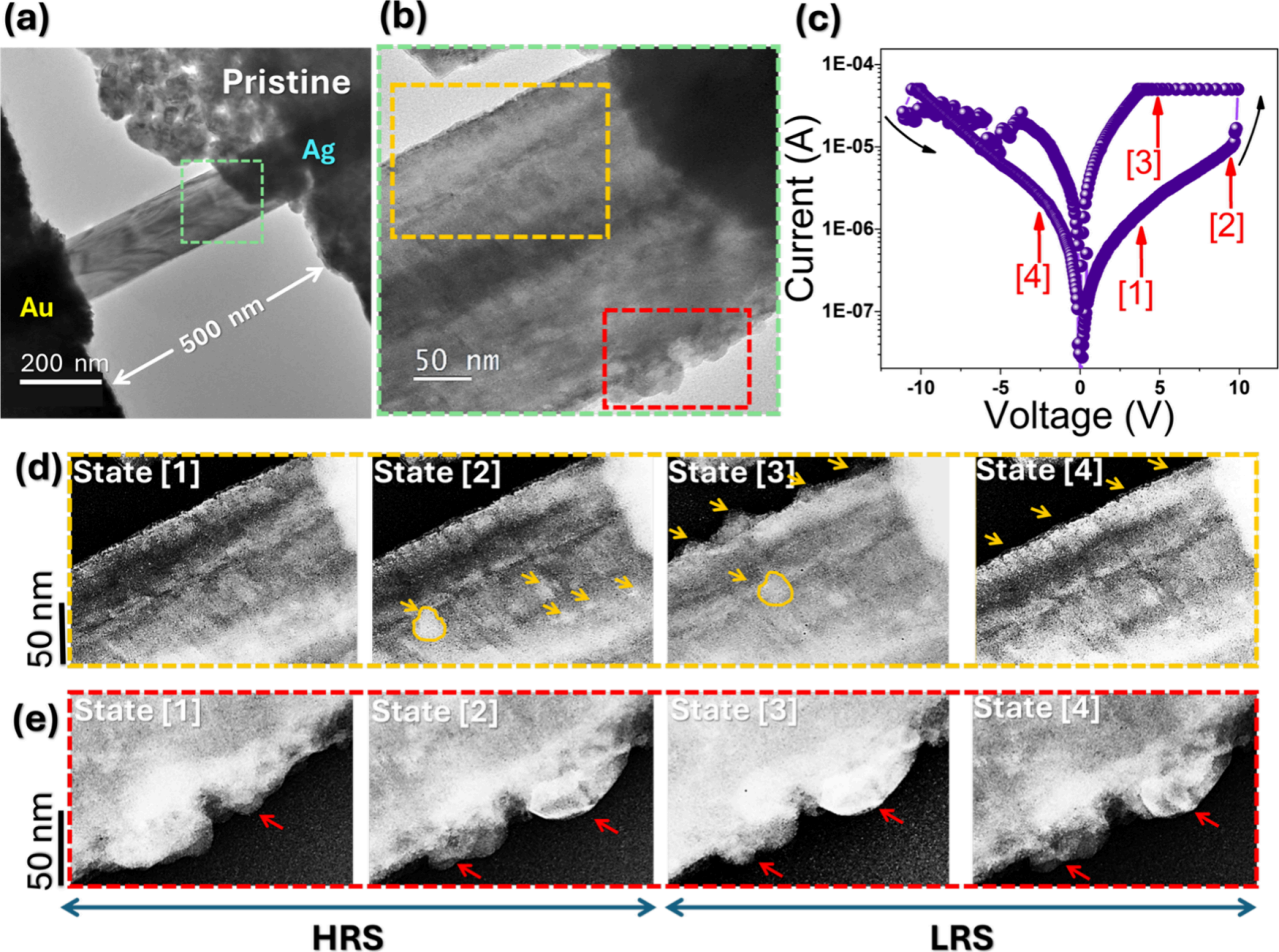
In situ TEM observations of an Ag/CuO NW/Au nonvolatile memristor under positive DC bias applied to the Ag electrode. (a) TEM images of the pristine Ag/CuO NW/Au device, featuring a NW diameter of 150 nm and tunneling gap distance of 500 nm. (b) An enlarged TEM image of the proximal region near the active Ag electrode (dashed green box in (a)). (c) *I*–*V* curve of the device showing the NVM process during in situ TEM observations. (d, e) Series of TEM images captured at different times, corresponding to the dashed yellow and dashed red boxes in panel (b), respectively. These images illustrate distinct states of the filament formation process, as indicated by the red arrows in panel (c). The arrows in the figure indicate the regions where Ag NPs dynamically evolve in response to the applied voltage.

The forming voltage is determined to be 9.8 V, marking the onset of RS behavior. The *I*–*V* curve initially shows a gradual increase in current up to the forming voltage followed by a sudden transition to the LRS, indicative of CF formation via the ECM process. In situ TEM imaging, corresponding to states 1 through 4 shown in Figure 5d,e, captures the dynamic evolution of the device structure at different voltages, illustrating a reversible switching between the HRS and LRS. This visual evidence directly reflects the voltage-driven filament growth and dissolution processes. The upper edge of the CuO NW displays a pristine surface with no observable Ag NPs at 0 V (State 1), as shown in Figure 5d. Upon reaching the forming voltage of 9.8 V (State 2), the greater signal contrast NPs in STEM image indicate higher atomic numbers of Ag NPs (highlighted by yellow arrows) (highlighted by yellow arrows) abruptly appear, marking the onset of filament formation. As the device enters the LRS (State 3), distinct Ag NPs (highlighted by yellow arrows) are observed along the upper edge of the NW, which continues to grow under sustained bias, reaching their maximum size. During the RESET process under negative bias, these Ag NPs begin to shrink and partially disappear as the voltage returns to 0 V (State 4), indicating the partial dissolution or fragmentation of the CFs and demonstrating a degree of reversibility in Ag accumulation. Interestingly, as shown in Figure 5e, additional Ag NPs nucleate along the lower edge of the NW during the LRS (indicated by red arrows). These particles grow prominently during State 2 and persist even after the bias is removed, suggesting incomplete dissolution and the possible retention of residual conductive pathways. Such remnants may act as localized conduction seeds, facilitating filament reformation and potentially lowering the SET voltage in subsequent cycles. The p-type CuO NWs used here possess a high density of intrinsic defects, both at the surface and within the bulk. This defect-rich nature is supported by prior observations: axial grain boundaries in HRTEM, spatial inhomogeneities in SAED patterns, and trap-mediated conduction mechanisms such as Poole–Frenkel emission and SCLC observed in electrical measurements. These defects serve as favorable sites for Ag^+^ ion trapping and NP nucleation during the switching process. Ag NPs dynamically grow and shrink in response to the applied bias, yet some persist at defect sites even after RESET, indicating their critical role as nucleation centers or intermediate states in filament formation. This behavior illustrates the importance of structural defects in governing Ag^+^ ion dynamics and the resulting RS characteristics. Although the complete continuous filamentary bridge exhibits weak contrast due to its ultrafine diameter and embedding within the CuO matrix, the formation of a conductive path is substantiated by the electrical transition to the LRS. Morphologically, this mechanism is supported by the direct observation of Ag NP nucleation and growth under bias. These Ag clusters serve as the discrete building blocks of the filament, providing indirect visual evidence of the Ag-based ECM process consistent with measured electrical switching. The presence of residual Ag NPs suggests that they serve as precursors for filament reconstruction, acting as a sustained Ag source during repeated SET/RESET operations. The progressive growth of NPs under increasing bias, along with their partial retention postbias, supports an ECM-based switching mechanism dominated by Ag^+^ ion migration and clustering. These findings provide compelling morphological evidence of filamentary ECM behavior in the absence of a visible, continuous Ag bridge, with charge transport likely occurring via short filament segments or hopping between adjacent NPs. Collectively, this insight into Ag NP dynamics highlights the fundamental role of electrochemical activity and defect-mediated ion transport in shaping the RS behavior of CuO NW-based memristors.

To further elucidate the relationship between Ag NP evolution and RS behavior, real-time in situ HAADF-STEM observations were conducted during electrical cycling. These experiments focused on monitoring the structural variations of Ag NPs in both the HRS and LRS, providing insight into their correlation with resistance changes. Figure S4 presents selected frames from an in situ HAADF-STEM video, analyzed using the Trainable Weka Segmentation (TWS) machine learning plugin for enhanced image classification. The segmented images distinguish four distinct regions: Ag NPs (red), CuO NWs (green), Ag electrode (purple), and background (yellow). Figure 6a presents the *I*–*V* characteristics under a compliance current of 1 μA, clearly demonstrating volatile RS behavior. Figure 6b displays an HAADF-STEM image of the CuO NW region adjacent to the active Ag electrode, with a magnified view and elemental mappings of Ag, Cu, and O, confirming that the bright contrast regions correspond to Ag deposits on the CuO NW surface, accumulated after repeated RS cycles. Notably, after 30 consecutive cycles, residual Ag NPs are clearly aligned along the NW axis, providing direct evidence of an ECM mechanism. In this process, Ag atoms from the active electrode undergo electrochemical dissolution, migrate as Ag^+^ ions, and are subsequently reduced and deposited onto the NW surface. Figure 6c presents time-sequenced STEM images capturing the real-time morphological evolution of Ag NPs under DC bias. These states correspond to the I–V profile shown in Figure 6a. Upon reaching the threshold voltage (*V*_th_≈ 10.5 V), Ag NPs undergo a clear transformation: from initial shape changes (States A–B), to gradual enlargement (State C), and then to partial shrinkage as the voltage returns to 0 V (States D–E). This behavior suggests incomplete NP dissolution and partial retention of conductive features. With increasing switching cycles, the postbias (0 V) density of residual Ag NPs increases markedly, becoming prominent after 100 cycles as shown in Figure S6. Co-registered STEM–EDS maps exhibit characteristic Ag signals at these sites, confirming their chemical identity as Ag residues. A representative HAADF–STEM image of the CuO NW device after 100 consecutive cycles is shown in Figure S6. Together with the corresponding EDS elemental maps, these are provided in Figure S7. Figure 6d summarizes the temporal evolution in both the number and average sizes of Ag NPs. During the LRS, the number of NPs decreases while their average size increases, indicating aggregation. This temporal correlation with the LRS further supports the notion that NP formation is critical in filament development. The illustrations in Figure 6e summarize the bias-driven evolution of Ag species inside the CuO NW observed by in situ TEM/STEM. **Step 1 (Initial state, HRS):** Prior to electrical stress, no discernible Ag clusters are detected inside the CuO NW by TEM, and the device remains in the HRS. In this regime, conduction is governed by hole transport through the p-type CuO matrix, evolving with field from Schottky emission to space-charge-limited conduction and then Poole–Frenkel type behavior; such carrier-transport processes do not produce strong mass–thickness contrast changes and are therefore not readily observable under TEM. **Step 2 (SET to LRS):** under a positive bias applied to the Ag electrode, Ag atoms at the active electrode are oxidized to mobile Ag species and injected into the CuO NW. Driven by the electric field, these Ag species drift into the NW and preferentially redistribute along the axial planar defect (TB), which serves as a low-barrier corridor for ion transport and accumulation. As bias increases, injected Ag species are reduced and nucleate into Ag NPs; continued supply leads to growth and aggregation of NPs and the development of a percolative metallic pathway, yielding an LRS. In our in situ TEM images, this transition is manifested by the emergence and clustering of Ag NPs. **Step 3 (RESET to HRS):** with residue: upon reversing the bias polarity, the metallic pathway becomes unstable and is dissolved/fragmented through electrochemical dissolution and local thermally assisted disruption, returning the device to the HRS. **Step 4 (After forming):** Importantly, a fraction of Ag NPs remains after RESET, functioning as residual nuclei that lower the nucleation barrier for the subsequent SET operation and thereby explaining the non-negligible Ag residue observed after cycling.

**Figure 6:**
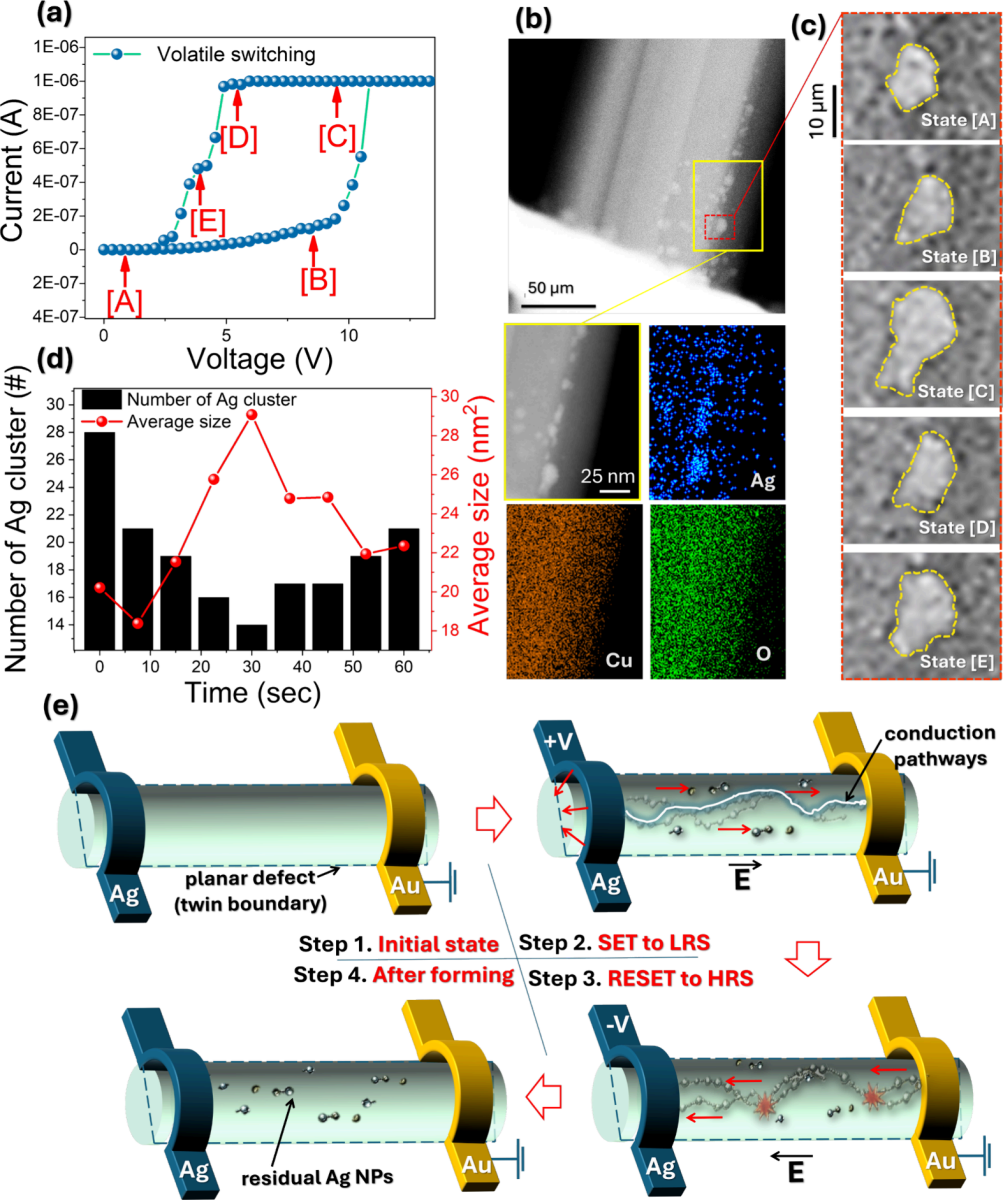
Accumulation phenomenon of Ag NPs after 30 consecutive cycles. (a) *I*–*V* curves at varying voltages, illustrating the ECM volatile switching process. (b) HAADF-STEM image of the region near the active Ag electrode with a magnified STEM image from the yellow box, the bright particles are the Ag precipitates, and corresponding EDS elemental mappings of Ag, Cu, and O elements, respectively. (c) A series of STEM images showing the structural evolutions of individual Ag NP states at different applied voltages. (d) Statistical analysis of the number and the average size of Ag NPs as a function of time. (e) Sequential schematic illustration of the NPs evolution. Step 1 (Initial state, HRS): Conduction is governed by hole transport (Schottky → SCLC → PF). Step 2 (SET to LRS): Under positive bias, Ag^+^ is injected, forming a conduction pathway. Step 3 (RESET to HRS): Under reverse bias, the conductive pathway ruptures due to electrochemical dissolution. Step 4 (After forming): Residual Ag NPs remain to lower the nucleation barrier for subsequent cycles.

Under sustained voltage, Ag^+^ ions originating from the electrode undergo repeated cycles of dissolution, migration, nucleation, and growth, resulting in the formation of CFs. Importantly, some Ag NPs persist after the voltage is removed, suggesting that they serve as stable nucleation centers that facilitate filament reformation and lower the SET voltage in subsequent switching cycles. These residual NPs enhance the local carrier transport by modifying nanoscale conductance pathways. Consequently, the interplay of Ag NP nucleation, growth, and partial dissolution underpins both volatile and nonvolatile switching behaviors in ECM-type memristive devices.

## 3 Conclusions

In conclusion, this study demonstrates the potential of p-type CuO NWs as a robust platform for memristive devices, leveraging their defect-rich structure and high aspect ratio to enable efficient resistive switching. In situ TEM/STEM analysis provides direct evidence of Ag^+^ ion migration and conductive filament formation, governed by electrochemical metallization in Ag/CuO NW/Au devices. The observed transition from volatile to nonvolatile switching, modulated by compliance current, highlights the tunability of RS behavior, while asymmetric electrode configurations induce diode-like characteristics due to Schottky barrier dynamics. The interplay of intrinsic defects, such as axial grain boundaries, with Ag nanoparticle nucleation underscores their critical role in facilitating filament growth and enhancing the switching stability. These findings elucidate the fundamental mechanisms underlying RS in one-dimensional nanomaterials and pave the way for their integration into high-density, low-power neuromorphic computing systems. Future research should focus on optimizing electrode materials and defect engineering to further enhance the device performance and scalability for practical applications.

## 4 Experimental Section

CuO NWs were synthesized using a thermal oxidation method, which provides a heady and scalable way to obtain oxide nanostructures. The experimental procedure is shown in Figure S1. Briefly, the Cu foam was used to increase the reaction surface area. The Cu foam was cut into 1 cm^2^ pieces, immersed in 1 M HCl for 2 min to remove native oxides, rinsed with deionized water, and dried at 40 °C for 1 h. The Cu foam was then placed in a furnace under ambient pressure and oxidized at 500 °C for 4 h; CuO NWs were grown vertically on the substrate accordingly. The devices were fabricated through lithographically patterned electrodes on an E-chip (Si_3_N_4_ membrane). The NWs were detached from the Cu foam and dispersed in acetone using an ultrasonicator for 3 min. The dispersion was drop-cast onto a homemade Si_3_N_4_ chip for in situ observation, as shown in Figure S4. In situ biasing TEM experiments were performed with a Protochips Fusion biasing holder and electrical E-chips incorporating prefabricated metal electrodes on a Si_3_N_4_ membrane window. Four built-in spring-loaded probes on the holder were contacted to the electrodes on the E-chip for the in situ TEM observations. The in situ TEM results data and electrical measurements were synchronized using commercial software (Protochips AXON). Photolithography employed a maskless digital light processing (DLP) system; the thicknesses of Au and Ag electrodes were both about 80 nm and were deposited with an e-beam evaporator as the inert and active electrodes, respectively.

This study employed CuO NWs with inherent defects to investigate the diffusion dynamics of Ag. In situ measurements were conducted using a JEOL ARM-200TH TEM at an accelerating voltage of 200 kV, equipped with a customized TEM holder (Fusion, Protochips), and a Keithley 2600B, Source Measure Unit (SMU) for simultaneous in situ TEM/STEM and electrical measurements. Real-time atomic-scale changes in the CuO NWs under electrical bias were captured, providing dynamic insights into material behavior during DC cycling and revealing the correlation among voltage, current, and atomic-scale dynamic transformations.

## Supplementary Material







## Notes

Images of n situ HAADF-STEM video using the Trainable Weka Segmentation (TWS) machine learning plugin for enhanced image classification (PDF, PDF)
Experimental procedures, SEM & TEM characterization of the CuO NWs, HAADF-STEM image and the results of corresponding EDS elemental mapping images with the atomic percentage of elements of CuO NW, learning workflow of the HAADF-STEM image segmentation via Trainable Weka Segmentation (TWS) machine learning technique (PDF)

## ABBREVIATIONS

RS: resistive switching
CuO NWs: cupric oxide nanowires
TEM: transmission electron microscopy
STEM: scanning TEM
HRTEM: high-resolution TEM
HAADF: high angle annular dark field
LRS: low-resistance state
HRS: high resistance state
ECM: electrochemical metallization
VCM: valence change mechanisms
AI: artificial intelligence
MIM: metal/insulator/metal
NVM: nonvolatile memories
VM: volatile memories
TB: twin boundary
XRD: X-ray diffraction
SAED: selected-area electron diffraction
VTH: threshold voltage
*I*_CC_: compliance current
SCLC: space- charge limited current
GBs: grain boundaries
CFs: conductive filaments
NPs: nanoparticles
SMU: source measure unit
