# Supplementary figures and images for: In Situ Scanning Transmission Electron Microscopy/Transmission Electron Microscopy Study of Defect-Driven Ag Ion Dynamics and Filament Evolution in CuO Nanowire-Based Memristors
Source: ACS Appl Mater Interfaces. 2026 Jan 5;18(1):2078–87. doi: 10.1021/acsami.5c21065 (PMC12781054; doi:10.1021/acsami.5c21065)

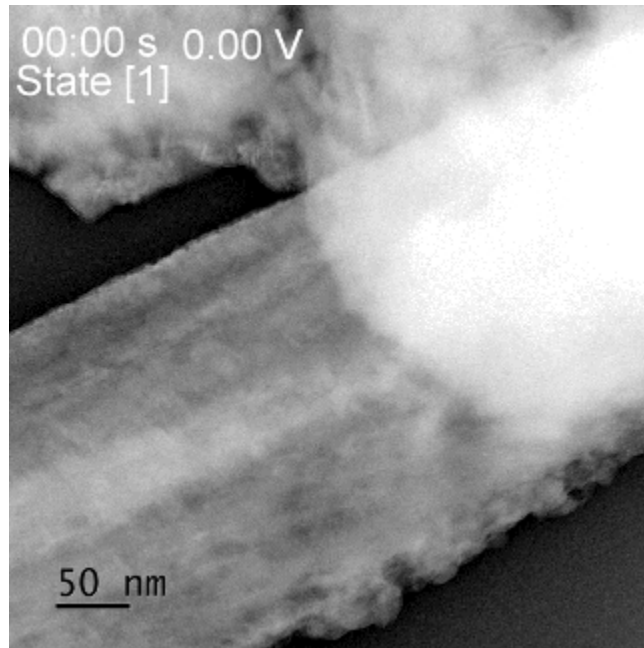

113x113mm (72 x 72 DPI)

Supplement: Supplementary file 1 [file am5c21065_si_001.pdf]

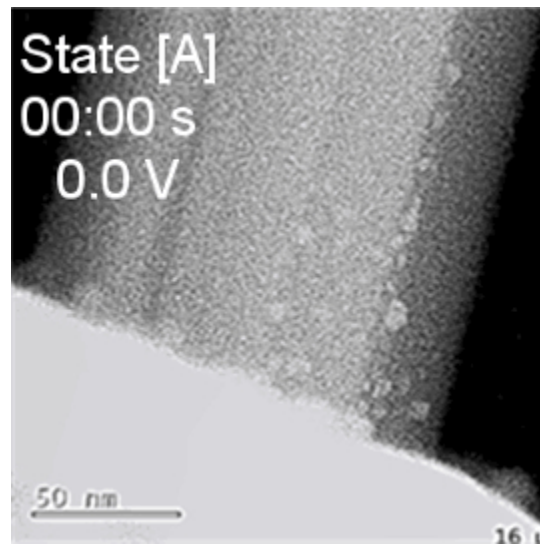

94x94mm (72 x 72 DPI)

Supplement: Supplementary file 2 [file am5c21065_si_002.pdf]
