# Supplementary material for: In Situ Scanning Transmission Electron Microscopy/Transmission Electron Microscopy Study of Defect-Driven Ag Ion Dynamics and Filament Evolution in CuO Nanowire-Based Memristors
Source: ACS Appl Mater Interfaces. 2026 Jan 5;18(1):2078–87. doi: 10.1021/acsami.5c21065 (PMC12781054; doi:10.1021/acsami.5c21065)
Supplement: Supplementary file 3 [file am5c21065_si_003.pdf]

Supporting Information

In-Situ STEM/TEM Study of Defect-Driven Ag Ion Dynamics and Filament Evolution in CuO Nanowire-Based Memristors

Ching-Heng Hung1, Chong-Chi Chi2, Kai-Yuan Hsiao1, and Ming-Yen Lu 1,2 *

1 Department of Materials Science and Engineering, National Tsing Hua University, Hsinchu 300, Taiwan

2 Instrumentation Center, National Tsing Hua University, Hsinchu 3000, Taiwan

*Corresponding Author: mylu@mx.nthu.edu.tw

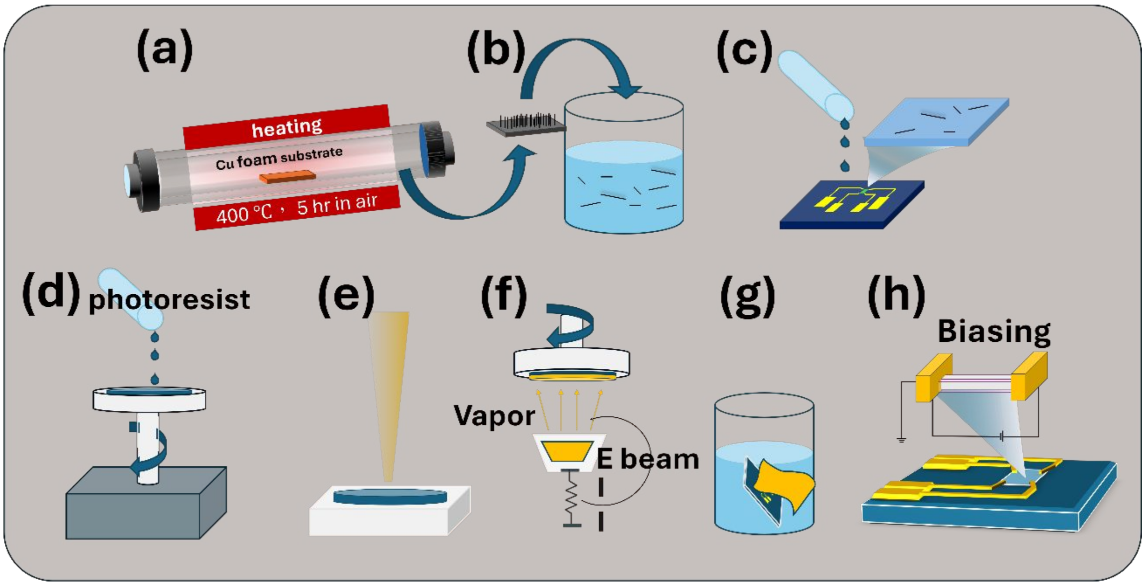

Figure S1. Fabrication of CuO NWs and Device Preparation(a) Thermally oxidation at 500°C for 4 hours. CuO NWs perpendicular to the Cu foil surface are obtained. (b) disperse the nanowires in the acetone solution. (c)The dispersed acetone solution is then drop-cast onto home-made Si_3N_4 chip. (d) photoresist is spin coated on the chip for (e)Maskless digital light processing (DLP). (f) electrodes are deposited with e-

beam evaporator. (g) lift off process and surface cleaning for (h) in-situ TEM observation.

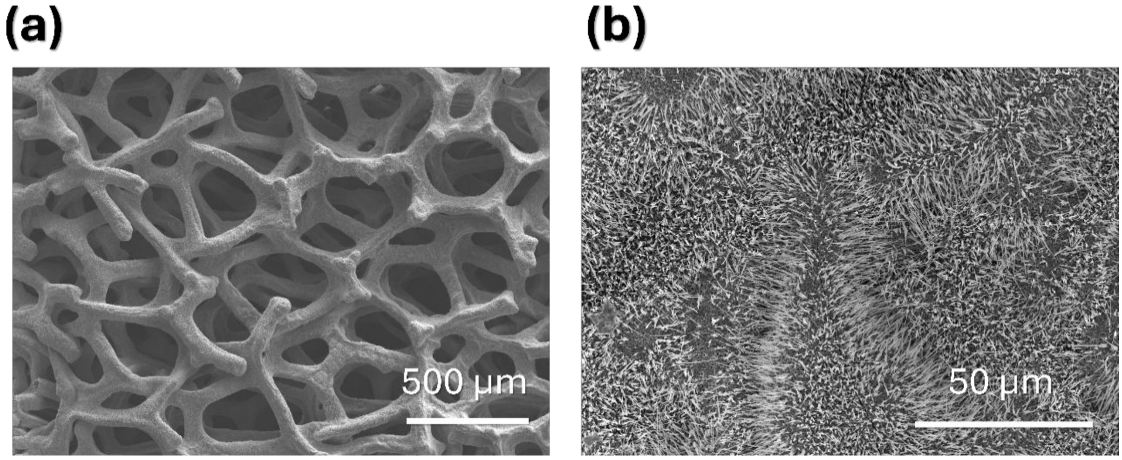

Figure S2. Additional SEM images of CuO NWs fabricate on (a) Cu foam and(b) low magnification SEM images of CuO NWs.

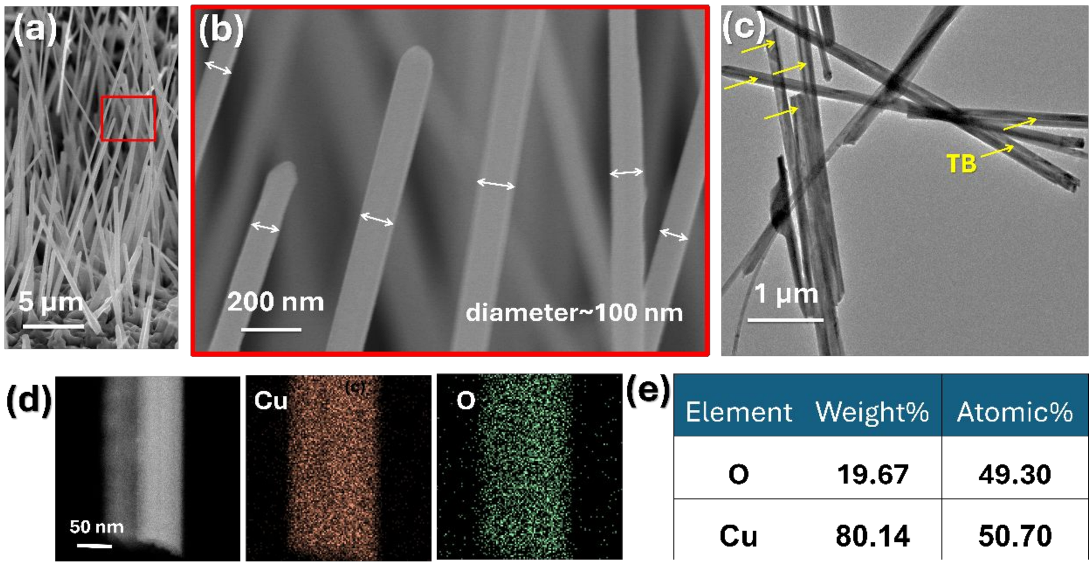

Element	Weight%	Atomic%
O	19.67	49.30
Cu	80.14	50.70

Figure S3. Morphological characterization of CuO NWs prepared via thermal oxidation, demonstrating a uniform morphology. SEM images at (a) low and (b) high magnifications, respectively, showing high density and uniform diameters (~100 nm). (c) TEM image of dispersed NWs; yellow arrows indicate axial TBs. (d) HAADF-STEM image and corresponding EDS elemental mappings of Cu, O. and (e) weight % and atomic % of the elements present in CuO NWs.

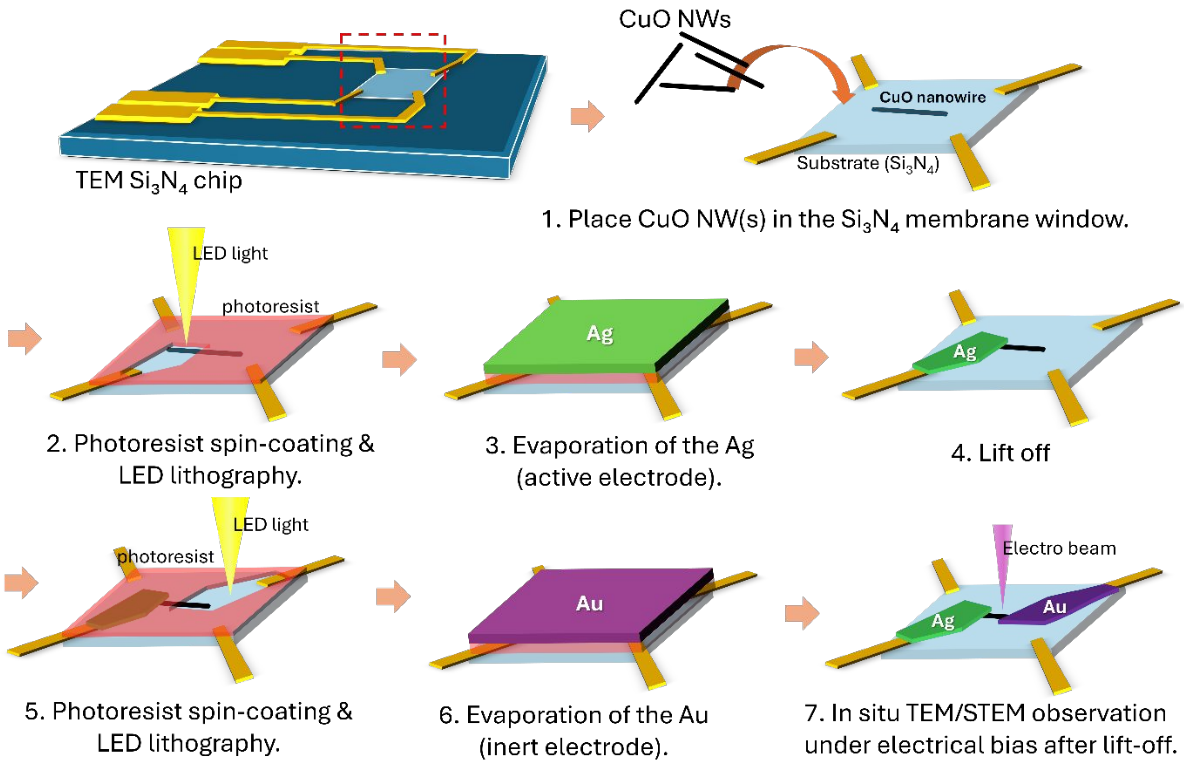

Figure S4. Step-by-step schematic of the device fabrication and electrical interfacing for in-situ TEM/STEM

Fabrication of TEM Si_3N_4 chip for in-situ TEM observation

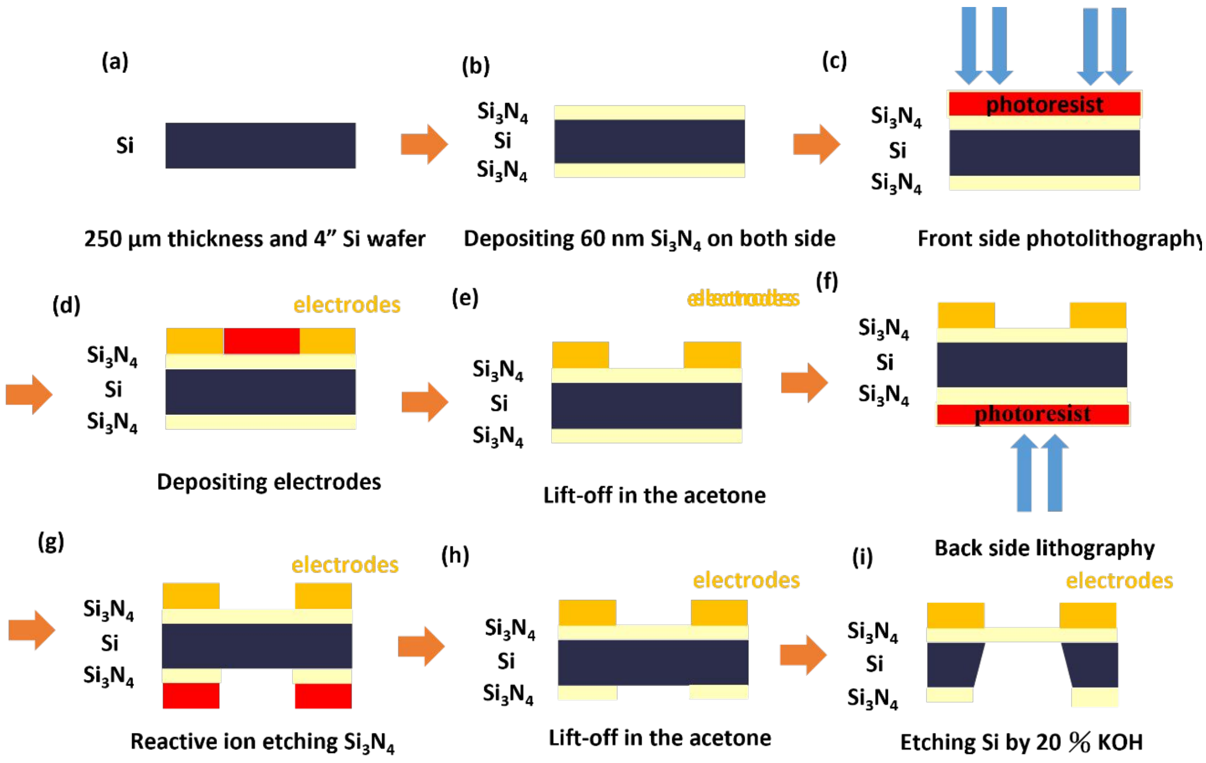

Figure S5. Step-by-step schematic of the TEM Si_3N_4 chip fabrication.

Briefly, (a) a Si wafer was used as the starting substrate; (b) both sides were nitrided to form dense, optically transparent Si_3N_4 membranes; (c) front-side photolithography defined metal pads and leads; (d) Cr (10 nm)/Au (80 nm) was deposited by e-beam evaporation to form the electrodes; (e) lift-off was performed in acetone with brief ultrasonication (≈30–60 s), followed by acetone/DI-water rinses to complete the front-side metallization; (f) photoresist was then spin-coated on the back side; (g) the backside Si_3N_4 was opened using a backside mask and RIE; (h) the resist was removed in acetone; and (i) the Si backside was wet-etched in 20 wt% KOH and the wafer was diced to yield chips compatible with the Fusion holder. CuO NWs were placed on the etched Si_3N_4 windows and Ag (active), and Au (inert) electrodes connected to the pre-patterned on-chip Au wiring, which interfaces directly with the holder contacts. I–V acquisition (Keithley SMU) and TEM/STEM imaging were performed simultaneously and synchronized.

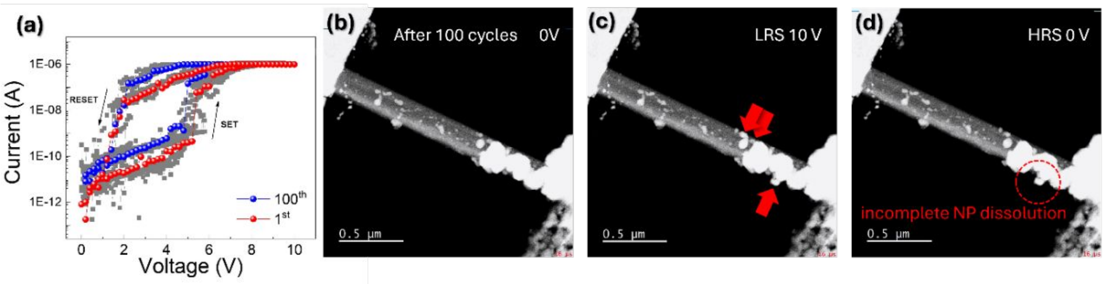

Figure S6. **(a)** The I-V curve and **(b)**HAADF-STEM image of the CuO NW device after 100 consecutive switching cycles, together with screenshots from the accompanying in-situ video showing the pristine, **(c)**LRS, and **(d)**HRS states.

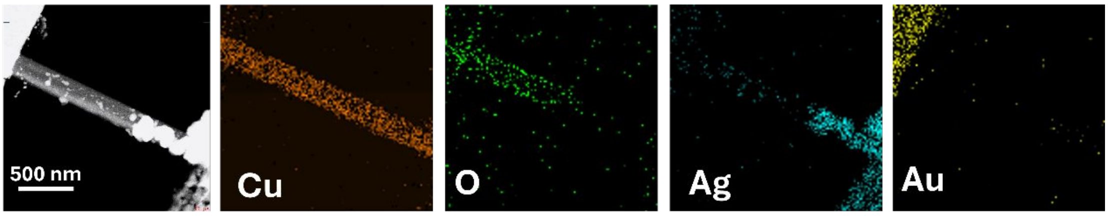

Figure S7. HAADF-STEM image of the CuO NW device after 100 consecutive cycles and corresponding EDS elemental mappings of Ag, Cu, and O elements, respectively.

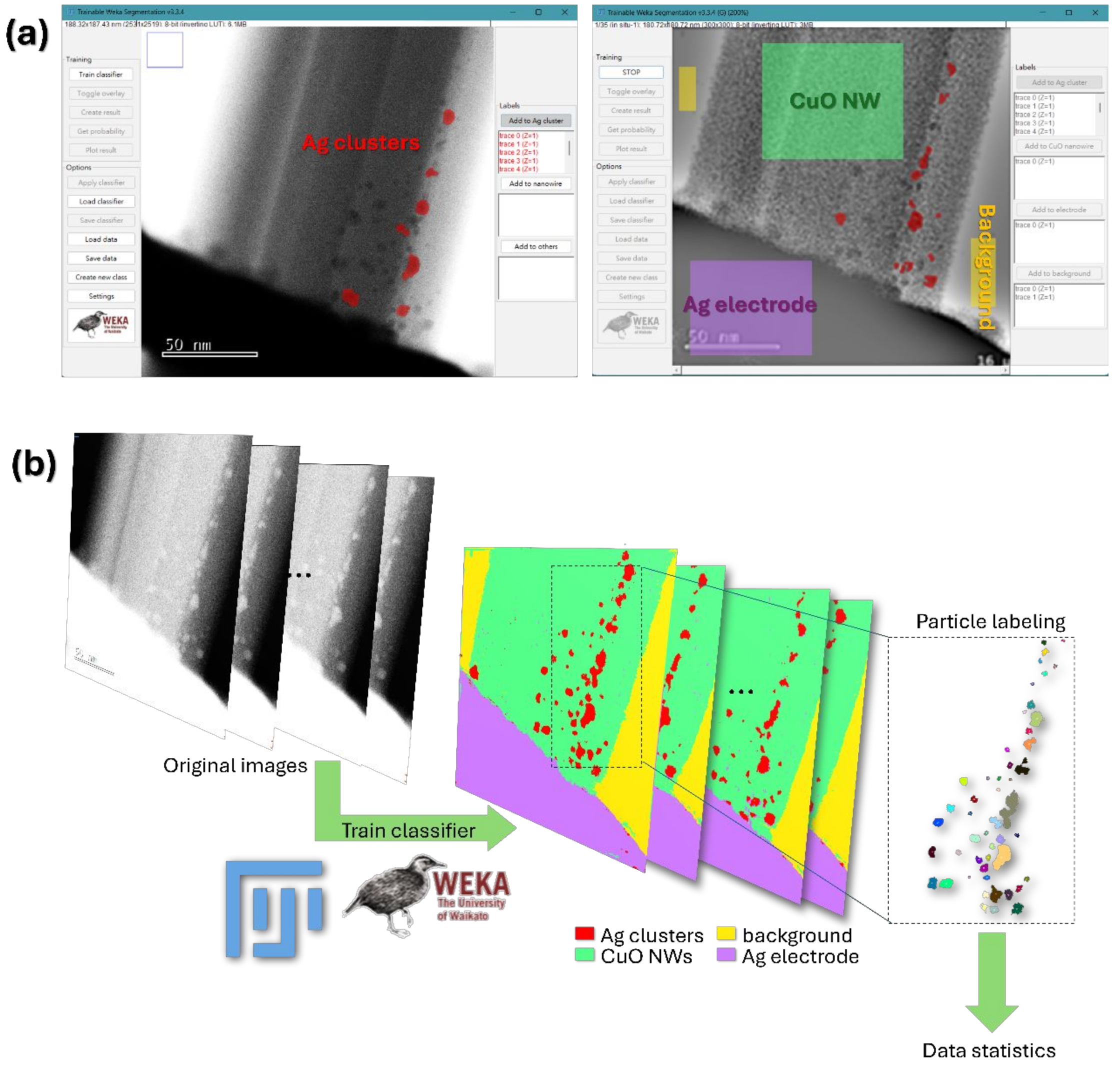

Fig. S8 The machine learning workflow of the HAADF-STEM image segmentation via Trainable Weka Segmentation (TWS) machine learning technique. (a) Training process, different regions corresponding to various materials are manually selected and labeled on the TEM images. (b) A series of snapshots of an in-situ HAADF-STEM video. Segmentation and quantification of snapshots by TWS. It is segmented into Ag nanoparticles (red), CuO NWs (green), Ag electrode (purple) and background(yellow).
